# Affiliate Stigma, Resilience and Quality of Life among Parents of Children with Autism Spectrum Disorder in Two Public Hospitals in Kelantan, Malaysia

**DOI:** 10.21315/mjms2024.31.3.17

**Published:** 2024-06-27

**Authors:** Noor Shuhada Salleh, Li Yoong Tang, Maruzairi Husain, Khatijah Lim Abdullah, Yee Cheng Kueh

**Affiliations:** 1 Nursing Programme, School of Health Sciences, Universiti Sains Malaysia, Kelantan Malaysia; 2 Department of Nursing Science, Faculty of Medicine, University of Malaya, Kuala Lumpur, Malaysia; 3 Department of Psychiatry, School of Medical Sciences, Universiti Sains Malaysia, Kelantan, Malaysia; 4 Department of Nursing, School of Medical and Life Sciences, Sunway University, Selangor, Malaysia; 5 Biostatistics and Research Methodology Unit, School of Medical Sciences, Universiti Sains Malaysia, Kelantan, Malaysia

**Keywords:** autistic disorder, child, parents, social stigma, quality of life

## Abstract

**Background:**

Often, the indirect impact of autism spectrum disorder (ASD) presents the family with significant challenges. One of these challenges is affiliate stigma due to parental affiliation with their child. This study aimed to explore affiliate stigma, resilience and quality of life (QoL) among parents of children with ASD.

**Methods:**

Cross-sectional study of 144 parents of children with ASD were recruited from two main tertiary hospitals in Kelantan, Malaysia, a developing country in Southeast Asia. Pearson correlation was used to examine the relationship between parental affiliate stigma, resilience and QoL. Simple and multiple linear regression analyses were used to identify the significant associated factors of affiliate stigma, resilience and QoL.

**Results:**

Correlational analyses revealed that perceived affiliate stigma demonstrated an inverse relationship with resilience and QoL. Additionally, resilience had a positive relationship with QoL. Regression analyses revealed that the father’s employment status, the mother’s level of education, having a disability card, the child’s age at ASD diagnosis, comorbidities of the child and ASD severity perceived by parents were associated with parental affiliate stigma, resilience and QoL.

**Conclusion:**

Study findings highlight the contribution of socio-demographic characteristics of children with ASD and their families in the determination of affiliate stigma, resilience and QoL.

## Introduction

Stigma is the adverse impact of negative attitudes or reactions towards individuals with mental health issues, in this case, a child with autism spectrum disorder (ASD). In addition, stigma also affects those directly involved or associated with them, such as family members. Parents of these individuals may experience stigma known as courtesy stigma due to their relationship (1).

**Figure 1:**
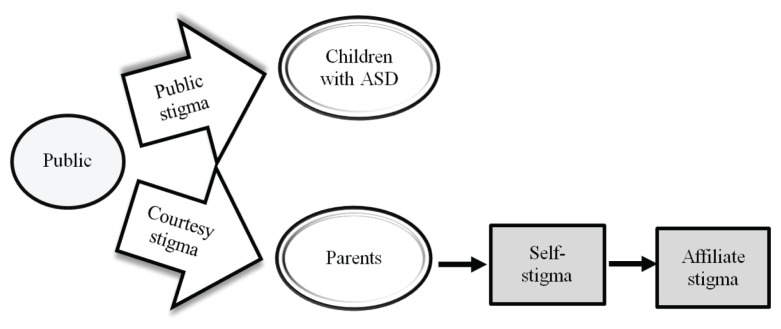
Stigma process between public, parents and children with ASD

Consequently, another type of stigma called affiliate stigma, involves internalisation of courtesy stigma toward the self and negative self-thought (Figure 1). Affiliate stigma damages self-esteem and has a substantial impact on mental health of parents of a child with ASD and, therefore, research on parents’ stigma internalisation is needed. Resilience is an intrinsic factor that can improve coping mechanism (2). Many parents of children with ASD have to deal with continual trials and challenges and are therefore usually resilient. Resilience helps maintain personal well-being and function (2). Parenting a child with ASD affects the family unit in terms of family members’ health and their quality of life (QoL) (3).

Research on the relationship between affiliate stigma, resilience and QoL is scarce (4). This study is designed to fill that gap. The aim was to explore parental affiliate stigma, resilience and QoL. This will inform nurse practitioners who play a critical role in identifying factors that contribute to affiliate stigma, which in turn affect parental resilience and, eventually, the family’s QoL. Hence, multifaceted interventions aimed at overcoming those factors could be provided efficiently. As a consequence, the improved QoL of parents will not only benefit the parental well-being, but also the child with ASD. Ultimately, an informed evidence-based study on these issues, as this current study is, is essential.

## Methods

### Study Design

This paper is part of a sequential explanatory mixed-methods study and it explores the quantitative part of the study rather than the qualitative design, which is discussed in previous research (4). This study was cross-sectional and included parents of children with ASD residing in Kelantan, a north-eastern state in Malaysia. Participants were recruited using convenience sampling when they accompanied their children for therapy at two tertiary public hospitals in Kelantan. Calculation in a study by Ozgur et al. (5) to determine the association between outcomes and predicted variables using GPower 3.1 was used to obtain the most conservative sample size (*n* = 85). An attrition rate of 10% was anticipated, hence, an additional nine participants were required (*n* = 94).

Eventually, a total of 144 participants were recruited. Inclusion criteria were: i) primary caregiver of at least one child with clinician-confirmed ASD diagnosis, in accordance with the Diagnostic and Statistical Manual of Mental Disorders, fifth edition (DSM-5) criteria (6); ii) major caregiving responsibility (including biological, adoptive/foster and step-parents); iii) living in the same residence as the child; iv) age of the child between 2 years old and 12 years old; and v) parental ability to read and comprehend Malay and/or English.

#### Measures

##### Affiliate Stigma Scale

The adapted version of the 12-item Affiliate Stigma Scale (ASS) by Zhou and colleagues was utilised (7). Psychometric properties of this scale have been evaluated and confirmed (8, 9), including excellent internal consistency (α = 0.80) (7). Item responses were coded using a 6-point Likert scale ranging from 1 = strongly disagree to 6 = strongly agree. Higher scores corresponded to higher levels of affiliate stigma. This scale was translated to Malay and validated among 30 parents of children with ASD in Kelantan. In the current study, α for the entire scale was 0.870.

##### Connor-Davidson Resilience Scale (10)

The 25-item Connor-Davidson Resilience Scale (CD-RISC) has been validated to measure an individual’s resilience (2, 10). Parents were asked to rate each item on how it described their feelings over the past month using a 5-point Likert-type scale ranging from 0 = not true at all to 4 = true nearly all the time. The highest possible total score was 100. Higher scores indicated a greater degree of perceived resilience. This scale was also translated into Malay and validated among 30 parents of children with ASD in Kelantan and demonstrated excellent internal consistency of 0.936.

##### Quality of Life in Autism-Parent Version (11)

The Quality of Life in Autism-Parent Version (QoLA-P) was utilised. It comprised 28 items and rated parents’ experiences during the past 4 weeks. The scale was measured on a 5-point Likert scale ranging from 1 = not at all to 5 = very much. Total scores ranged from 28 to 140 with higher scores corresponding to better perceived QoL. The QoLA-P had excellent internal consistency (α = 0.940) and strong concurrent validity with scores on all four subscales of the WHOQoL-BREF (*r* = 0.74–0.91, *P* < 0.01) (11). The original validation study was conducted in Malaysia to assess QoL among parents of a child with ASD (12).

### Statistical Analysis

Data analyses were performed using the Statistical Package for Social Sciences (SPSS) version 22.0. There was no missing data as the researcher ensured that all items were answered during data collection. Parametric tests were used as data was normally distributed. Pearson’s correlation was used to examine the relationship between affiliate stigma, resilience and QoL (correlation was significant at *P* = 0.01 [2-tailed]). Variables with *P* < 0.25 upon simple linear regression or those that were clinically important were included in a multiple linear regression model. Automated variable selection used in this analysis included stepwise, backward and forward methods. Results derived from these methods were evaluated and compared. Independent variables with a *P*-value < 0.05 were included in the preliminary main effect model. Assumptions of multiple linear regression were then checked: i) no multicollinearity; ii) interaction between each independent variable was checked. If *P* > 0.05, the interaction was considered not significant; iii) overall linearity and equal variance were checked via a scatter plot; and iv) normality was checked using a histogram. When all assumptions were met, the final multiple linear regression model was identified.

## Results

### Parents’ Characteristics

Respondents’ ages ranged from 23 years old to 54 years old (M = 36.4, SD = 5.78). Sixty-two parents (43%) (female [F] = 15, male [M] = 47) were government sector employees, 23 (16%) (F = 13, M = 10) were private sector employees, 18 (13%) (F = 5, M = 13) were self-employed. All fathers were employed while, 41 out of the 111 mothers were homemakers (37%) (Table 1).

### Family Characteristics

Median monthly family income was RM3,001–RM5,000 which was consistent with the median income in urban areas of Kelantan (RM4,019) but lower than the national median income (RM5,873) (13). A majority (89%) of the families comprised multiple children, with two being the most common.

### Child’s Characteristics

The male to female ratio was 4.8:1; consistent with a study in 2016 which estimated a male to female ratio of 3.6–5.1 to 1 (14). All (100%) of the parents reported that their children had at least one ‘other condition’ associated with ASD, most frequently (29.9%) two additional conditions (range = 1–5). The conditions listed were consistent with those typically cited in ASD literature (Table 2).

### Mean Scores of Parental Affiliate Stigma, Resilience and QoL

Total mean scores were: affiliate stigma, 25.01 (SD = 9.28); resilience, 73.86 (SD = 13.41) and QoL, 103.76 (SD = 15.96).

### Relationship between Parental Affiliate Stigma, Resilience and QoL

Affiliate stigma had a statistically significant inverse relationship with resilience and QoL (*r* = −0.51, *P* < 0.001; *r* = −0.55, *P* < 0.001, respectively). Higher affiliate stigma was associated with poorer resilience and poorer QoL. Conversely, resilience had a statistically significant positive relationship with QoL (*r* = 0.75, *P* < 0.001). Thus, higher resilience was associated with higher QoL.

### Sociodemographic Characteristics Associated with Affiliate Stigma, Resilience and QoL

There were five significant factors associated with affiliate stigma, three associated with resilience and four with QoL (Table 3).

The final model equation of affiliate stigma was: 15.18 + (4.63 × father’s employment status, private sector worker) + (5.43 × mother’s educational level, degree/Masters/PhD) + (3.69 × disability card holder) − (1.22 × child’s age at diagnosis of ASD) + (1.42 × ASD severity perceived by parents).

The final model equation of resilience: 88.85 − (4.56 × disability card holder) − (7.38 × comorbidities of the child) − (2.35 × ASD severity perceived by parents).

The final model equation of QoL: 125.89 − (9.37 × father’s employment status, private sector worker) − (6.29 × disability card holder) − (9.26 × comorbidities of the child) − (2.80 × ASD severity perceived by parents).

Variance resulted in regression (R^2^) ranging from 20.2% for resilience to 27.0% for QoL. This suggests that there were other sociodemographic characteristics that influenced the score but had not been examined.

## Discussion

The mean ASS score (25.01) was remarkably lower than in the study conducted by Shin et al. (15) among Malaysian parents of children with ASD (38.12). This may be due to different sociodemographic backgrounds as Shin et al. (15) recruited 110 parents located in Kuala Lumpur. Even so, we acknowledge that raising a child with ASD is not a uniform experience as various factors may influence the level of perceived stigma. The mean ASS score in this study was also much lower than reported by Zhou et al. (7) in China (45.02). This may be due to previous studies which found that Chinese parents appear to suffer from higher stigma as they are more likely to focus on their social identity, value and saving face (7, 16). However, the majority of parents in our study strongly disagreed that ‘*Having a child with ASD makes me lose face*’ (*n* = 97, 67.4%). This showed that they were not ashamed of having a child with ASD and therefore reported lower affiliate stigma. This discrepancy may be due to the difference in cultural acceptance of disability.

The mean CD-RISC score was 73.86; higher than two other studies: for Bitsika et al. (2), the CD-RISC score among 108 parents was 66.37 and for Whitehead et al. (17) the score was 62.83 for 438 parents of children with ASD. Differences in resilience scores may be explained by different coping strategies employed by parents in Asian countries (15), as culture plays an important role in the use of parental coping strategies. ‘*When there are no clear solutions to my problems, sometimes faith or God can help*’ was the most agreed upon statement among parents when describing their resilience (*n* = 105, 72.9%). Religious belief played a relatively large role among parents in this study, compared to studies carried out in a Western/European context (18, 19).

The mean QoLA-P score (103.76) was higher than for Due et al. (20) and Eapen et al. (11). However, it is consistent with Isa et al. (21) which reported higher QoL scores in Malaysian parents. This may be part of the local culture’s tendency to generate positive responses in maintain an expected ‘perfect’ identity (21). Also, higher QoL may be closely related to higher resilience which was found among parents in this study. This is supported by studies with similar findings which have indicated that successful adaptation to adversity or resilience can further enhance parental QoL (22, 23).

### Relationship between Affiliate Stigma, Resilience and QoL

Affiliate stigma was significantly and inversely related to resilience. This is consistent with other studies (7, 22). Thus, parental affiliate stigma has a negative impact on resilience and QoL.

There was a strong, statistically significant positive relationship between resilience and QoL, similar to other studies (17, 22). Resilience is a vital proxy support system and essential health resource for improved parental QoL to enable parents to have a positive health status. Thus, policymakers should invest in parenting skills training programmes for this group.

### Sociodemographic Characteristics and Affiliate Stigma

Fathers who worked in private sector experienced higher affiliate stigma. In Malaysia, government-sector wages are higher than private-sector wages in most sectors (24). Thus, fathers working in the private sector have may lower economic reserves. Additionally, having a child with ASD may be associated with significant additional healthcare and non-medical expenses (25). Limited income may prevent them from obtaining the resources they need and thus may increase affiliate stigma.

Mothers with higher educational qualifications perceived more affiliate stigma. A higher parental level of education has been documented to be associated with earlier problem recognition (26). Hence, one possible reason for our new finding is that mothers with a higher educational status may potentially be exposed to repeated instances of stigmatising situations during care-seeking. This would result in more protracted perceived stigma.

Parents whose child had a disability card, had higher affiliate stigma. Higher stigma is found in collectivistic cultures compared to individualistic cultures, possibly due to less acceptance of cultural diversity in in the former (27, 28). Therefore, having a disability card may potentially marginalise the child.

Older age of the child at diagnosis lowered affiliate stigma by 1.22 units, indicating that parents perceived lower stigma if their child was diagnosed at an older age. Parents may be more likely to face negativism and challenges in making sense of their child’s difficulties at earlier ages, as stigma may have been more intensely experienced. This is consistent with a study which showed that parents of a child with newly diagnosed ASD struggle more with stigma (27).

Parents who perceived their child to have greater ASD severity also perceived more stigma, consistent with past research (9). This may be because parents perceived greater stigma when their child exhibited more autism-related behaviours, which were often perceived as shameful and embarrassing.

### Sociodemographic Characteristics and Resilience

Parents of a child with a disability card, comorbidities and higher perceived ASD reported lower resilience. The social impact of having a disability card may lower resilience especially in Asian cultures, where there tends to be an emphasis on social identity and value (7, 16).

Co-morbidities and more severe ASD symptoms reduced parental resilience. Parents of children with ASD may have a higher burden and therefore lower levels of resilience (10).

### Sociodemographic Characteristics and QoL

QoL was influenced by several sociodemographic factors. Fathers who worked in the private sector had lower QoL scores. This may be due to reduced parental capacity to make positive changes in their environments due to limited resources, hence, lowering QoL (29).

In Malaysia, facilities available to children with ASD are limited in rural areas (30). Thus, parents at times seek treatment from private services. This situation could lead to cumulatively high financial demands on families; thereby influencing their QoL. Having a disability card was associated with lower parental QoL. Again, cultural views may be the reason behind this. Parents whose child had co-morbidities and more severe ASD symptoms also had lower QoL. This is supported by other literature (5, 31).

This study was based on a predominantly ethnic Malay sample (*n* = 95.8%). Kelantan is home to a majority population of Malay Muslims (95%) and a small group of other ethnic groups such as Chinese (3.4%) and other minorities such as Thais, Orang Asli (native people) and Indians (32). This may limit the generalisability of the findings. However, it can explain the needs of the Malay population in Kelantan and possibly other states that share a similar ethnic distribution.

Also, the study was conducted at 2 of the 10 general hospitals in Kelantan, Malaysia. Hence, these results may not be fully representative of the care experiences of parents of children with ASD throughout the state. This study did not involve parents whose children attended private centres or those who left or defaulted intervention. Future research should involve parents from wider geographical and socio-demographic backgrounds in other states in Malaysia and Southeast Asia to determine whether their experiences mirror what is reported in the study.

The majority of participants were mothers (*n* = 77.1%). Nonetheless, this reflects the distribution of parental responsibilities within many families. This gender imbalance was also because the study examined primary caregivers, who were mothers in most cases. Future studies should explore potential differences between mothers’ and fathers’ parenting experiences.

Ideally, the age range should include adolescents and adults with ASD. This would provide insight into more diverse experiences of parenting. The majority of the children with ASD in this study were boys (82.6%). Future research should focus on the effect of the children’s gender on parents’ experiences.

This study provides a snapshot of affiliate stigma, resilience and QoL at one point in time. However, these experiences are expected to fluctuate over time. Longitudinal research that adopts a parental life cycle perspective will add critical insight.

## Conclusion

In summary, this study allowed us to create a better understanding of the perceived affiliate stigma, resilience and QoL among parents of children with ASD. Despite its limitations, it represents the first known detailed exploration of parental affiliate stigma, resilience and QoL in Malaysia, therefore this study may provide preliminary evidence applicable to Malaysian parents in need of social and mental healthcare support. In other words, services should be tailored to address the support needs of parents. Policymakers and nurse practitioners directly or indirectly involved in providing care for children with ASD need to include and meet parental needs as part of holistic management to improve overall care. Current nurse practitioners may be partly aware of the affiliate stigma perceived by parents and its effect on their resilience and QoL. Nevertheless, the recognition and contribution of sociodemographic characteristics in the determination of affiliate stigma, resilience and QoL is still underappreciated. Hence, this study will be an essential starting point towards creating an effective standard of practice among nurse practitioners.

## Figures and Tables

**Table 1:** Socio-demographic characteristics of parents and families (*N* = 144)

Demographic information	*n* (%)
Relationship to child with ASD	
Mother	111 (77.1)
Father	33 (22.9)
Family ethnicity	
Malay	138 (95.8)
Chinese	5 (3.5)
Indian	0 (0)
Others a	1 (0.7)
Marital status	
Married	136 (94.4)
Divorced	5 (3.5)
Widowed	2 (1.4)
Never married	1 (0.7)
Father’s employment status	
Government sector worker	61 (42.4)
Private sector worker	40 (27.8)
Self-employed	41 (28.5)
Retiree	1 (0.7)
Not applicable	1 (0.7)
Mother’s employment status	
Government sector worker	57 (39.6)
Private sector worker	16 (11.1)
Homemaker	57 (39.6)
Self-employed	14 (9.7)
Father’s educational level	
Primary school	2 (1.4)
Secondary school	45 (31.3)
Higher school certificate/Diploma	46 (31.9)
Degree	42 (29.2)
Masters/PhD	8 (5.6)
Not applicable	1 (0.7)
Mother’s educational level	
Secondary school	33 (22.9)
Higher school certificate/Diploma	45 (31.3)
Degree	51 (35.4)
Masters/PhD	15 (10.4)
Member of ASD support group	
Yes	78 (54.2)
No	66 (45.8)
Participated in any activity organised by support group	
Yes	94 (65.3)
No	50 (34.7)
Monthly family income b	
RM1,000 and less	7 (4.9)
RM1,001–RM2,000	21 (14.6)
RM2,001–RM3,000	33 (22.9)
RM3,001–RM5,000	22 (15.3)
RM5,001–RM8,000	32 (22.2)
RM8,001 and above	29 (20.1)
Age of siblings in the family	
Less than 1 years old	15 (10.4)
1 year old–6 years old	111 (77.1)
7 years old–12 years old	81 (56.3)
13 years old–17 years old	27 (18.8)
18 years old and above	7 (4.8)
Multiple children with disabilities	
Yes	10 (6.9)
No	134 (93.1)
Sibling with diagnosed disabilities	
ASD	7 (70.0)
ASD + ADHD c	1 (10.0)
Dyslexia	1 (10.0)
Down syndrome	1 (10.0)

Notes:

a The ‘Others’ category under ethnicity included Siamese (*n* = 1). As one mother (*n* = 1) met the ‘Never married’ category, there led to the ‘Not applicable’ category under the father’s employment status and educational level, due to the lack of a partner;

b RM4.65 = 1 USD;

c ADHD = attention-deficit hyperactivity disorder

**Table 2:** Socio-demographic characteristics of the child (*N* = 144)

Demographic information	*n* (%)	Mean (SD)
Age of child (years old)		6.17 (2.75)
Age at diagnosis of ASD (years old)		3.30 (1.24)
ASD severity perceived by parents a		4.83 (1.82)
Gender		
Male	119 (82.6)	
Female	25 (17.4)	
Type of school		
Not yet started	25 (17.4)	
Playschool/nursery	66 (45.8)	
Special needs class	49 (34.0)	
Mainstream class	2 (1.4)	
Not schooling	2 (1.4)	
Receiving a disability allowance		
Yes	56 (38.9)	
No	88 (61.1)	
Disability card holder		
Yes	90 (62.5)	
No	54 (37.5)	
Comorbidities		
Yes b	15 (10.4)	
No	129 (89.6)	
Presence of other conditions associated with ASD		
Hyperactivity	51 (35.4)	
Speech and/or language difficulties	119 (82.6)	
Sleep difficulties	32 (22.2)	
Learning difficulties	91 (63.2)	
Feeding difficulties	76 (52.8)	
Others c	10 (69.4)	

Notes:

a Parents being asked regarding ASD severity using a one to ten scale on how problematic the child was on a daily basis;

b ‘Comorbidities’ included asthma (*n* = 7), epilepsy (*n* = 2), asthma and epilepsy (*n* = 1), hearing impairment (*n* = 2), vision impairment (*n* = 1), heart problems (*n* = 1), and thalassemia (*n* = 1);

c ‘Others’ conditions associated with ASD included social and behavioural problems (*n* = 7), sensory integration impairment (*n* = 2) and mood/emotion/expression problems (*n* = 1)

**Table 3:** Factors associated with affiliate stigma, resilience and QoL in parents of children with ASD (*N* = 144)

Variables	Simple linear regression		Multiple linear regression		

	Crude *b*^a^ (95% CI)	*P*-value	Adj. *b*^b^ (95% CI)	*t*-stat	*P*-value
Factors associated with affiliate stigma					

Father’s employment status					
Government sector worker*	0		0		
Private sector worker	3.577 (−0.13, 7.86)	0.058	4.631 (1.21, 8.05)	2.678	0.008
Self-employed	−0.284 (−3.91, 3.34)	0.877	1.672 (−1.68, 5.02)	0.987	0.326
Mother’s educational level					
Secondary school*	0		0		
Higher school certificate/Diploma	0.515 (−3.61, 4.64)	0.806	1.359 (−2.44, 5.16)	0.707	0.481
Degree/Masters/PhD	4.424 (0.58, 8.27)	0.024	5.427 (1.83, 9.02)	2.985	0.003
Disability card holder					
No*	0				
Yes	4.396 (1.31, 7.48)	0.006	3.692 (0.72, 6.66)	2.458	0.015
Child’s age at diagnosis of ASD	−0.973 (−2.21, 0.26)	0.122	−1.218 (−2.34, −0.10)	−2.148	0.033
ASD severity perceived by parents	1.680 (0.88, 2.48)	< 0.001	1.416 (0.64, 2.20)	3.596	< 0.001
Coefficients of determinants, R^2^ = 24.7%, *F* = 6.37					

Factors associated with resilience					

Disability card holder					
No*	0				
Yes	−7.511 (−11.92, −3.11)	0.001	−4.558 (−8.86, −0.26)	−2.095	0.038
Comorbidities of the child					
No*	0				
Yes	−9.743 (−16.82, −2.67)	0.007	−7.375 (−14.01, −0.75)	−2.199	0.030
ASD severity perceived by parents	−2.752 (−3.89, −1.62)	< 0.001	−2.353 (−3.49, −1.22)	−4.099	< 0.001
Coefficients of determinants, R^2^ = 20.2%, *F* = 11.81					

Factors associated with QoL					

Father’s employment status					
Government sector worker*	0		0		
Private sector worker	−8.602 (−14.90, −2.30)	0.008	−9.370 (−15.03, −3.71)	−3.276	0.001
Self-employed	−2.506 (−8.67, 3.66)	0.423	−3.676 (−9.18, 1.82)	−1.322	0.188
Disability card holder					
No*	0				
Yes	−8.822 (−14.07, −3.57)	0.001	−6.294 (−11.28, −1.30)	−2.494	0.014
Comorbidities of the child					
No*	0				
Yes	−11.643 (−20.06, −3.23)	0.007	−9.263 (−16.88, −1.65)	−2.405	0.018
ASD severity perceived by parents	−3.436 (−4.77, −2.10)	< 0.001	−2.799 (−4.11, −1.49)	−4.232	< 0.001
Coefficients of determinants, R^2^ = 27.0%, *F* = 10.20					

Notes:

a Crude regression coefficient;

b Adjusted regression coefficient;

* Reference variable; Stepwise, backward and forward multiple linear regression method applied. Model assumptions were fulfilled. There were no interactions amongst independent variables. No multicollinearity detected
